# Initial Experience of Challenge-Free MRI-Based Oxygen Extraction Fraction Mapping of Ischemic Stroke at Various Stages: Comparison With Perfusion and Diffusion Mapping

**DOI:** 10.3389/fnins.2020.535441

**Published:** 2020-09-16

**Authors:** Shun Zhang, Junghun Cho, Thanh D. Nguyen, Pascal Spincemaille, Ajay Gupta, Wenzhen Zhu, Yi Wang

**Affiliations:** ^1^Department of Radiology, Tongji Hospital, Tongji Medical College, Huazhong University of Science and Technology, Wuhan, China; ^2^Department of Radiology, Weill Cornell Medicine, New York, NY, United States; ^3^Department of Biomedical Engineering, Cornell University, Ithaca, NY, United States

**Keywords:** ischemic stroke, MRI, oxygen extraction fraction, cerebral metabolic rate of oxygen, quantitative susceptibility mapping, cerebral blood flow

## Abstract

MRI-based oxygen extraction fraction imaging has a great potential benefit in the selection of clinical strategies for ischemic stroke patients. This study aimed to evaluate the performance of a challenge-free oxygen extraction fraction (OEF) mapping in a cohort of acute and subacute ischemic stroke patients. Consecutive ischemic stroke patients (a total of 30 with 5 in the acute stage, 19 in the early subacute stage, and 6 in the late subacute stage) were recruited. All subjects underwent MRI including multi-echo gradient echo (mGRE), diffusion weighted imaging (DWI), and 3D-arterial spin labeling (ASL). OEF maps were generated from mGRE phase + magnitude data, which were processed using quantitative susceptibility mapping (QSM) + quantitative blood oxygen level-dependent (qBOLD) imaging with cluster analysis of time evolution. Cerebral blood flow (CBF) and apparent diffusion coefficient (ADC) maps were reconstructed from 3D-ASL and DWI, respectively. Further, cerebral metabolic rate of oxygen (CMRO_2_) was calculated as the product of CBF and OEF. OEF, CMRO_2_, CBF, and ADC values in the ischemic cores (absolute values) and their contrasts to the contralateral regions (relative values) were evaluated. One-way analysis of variance (ANOVA) was used to compare OEF, CMRO_2_, CBF, and ADC values and their relative values among different stroke stages. The OEF value of infarct core showed a trend of decrease from acute, to early subacute, and to late subacute stages of ischemic stroke. Significant differences among the three stroke stages were only observed in the absolute OEF (*F* = 6.046, *p* = 0.005) and relative OEF (*F* = 5.699, *p* = 0.009) values of the ischemic core, but not in other measurements (absolute and relative CMRO_2_, CBF, ADC values, all values of *p* > 0.05). In conclusion, the challenge-free QSM + qBOLD-generated OEF mapping can be performed on stroke patients. It can provide more information on tissue viability that was not available with CBF and ADC and, thus, may help to better manage ischemic stroke patients.

## Introduction

Ischemic stroke due to impaired blood flow to the brain is one of the leading causes of mortality and morbidity all over the world (Benjamin et al., 2017; Wang et al., 2017). A major therapy objective is to salvage tissue in the ischemic penumbra, a region with perfusion below a functional threshold but above a preservation threshold (Dirnagl et al., 1999; Pushie et al., 2018; Thirugnanachandran et al., 2018). The penumbra is estimated using MRI according to the mismatch between perfusion weighted imaging (PWI, indicating a functional threshold) and diffusion weighted imaging (DWI, non-hyperintensity indicating a preservation threshold). However, the difficulty in perfusion quantification makes it problematic to define a PWI threshold (Wouters et al., 2017; Zaro-Weber et al., 2017). The PWI–DWI mismatch may overestimate the penumbral tissue with the mismatch volume varying with quantification methods (Sobesky et al., 2005).

Penumbra evolves rapidly within the first few hours (Dirnagl et al., 1999; Pushie et al., 2018; Thirugnanachandran et al., 2018), and 24 h may be the threshold time window beyond which ischemic lesion becomes irreversible (Bonova et al., 2013). Accordingly, therapy of ischemic stroke is guided by the time from stroke onset. In current guidelines, intravenous administration of thrombolytic tissue plasminogen activator can be performed within 4.5 h (Davis and Donnan, 2009), and endovascular thrombectomy guided by advanced imaging of penumbral pattern can be performed within 24 h (Saver et al., 2016; Albers et al., 2018; Nogueira et al., 2018; Powers et al., 2018; Adeoye et al., 2019). Additionally, about 14% of strokes are wake-up types without known onset time (Mackey et al., 2011). Therefore, it is important for stroke therapy to assess tissue viability in ischemic lesions and differentiate stroke stages (Allen et al., 2012).

Oxygen extraction fraction (OEF) mapping reflects tissue metabolic state and is regarded as a very sensitive parameter in characterizing neural damage as tissue evolves from oligemia, to penumbra, and finally to death during ischemia (Powers, 1991; Leigh et al., 2018). The ^15^O positron emission tomography (^15^O-PET) is the gold standard for quantitatively assessing OEF and cerebral metabolic rate of oxygen (CMRO_2_). However, the 2 min half-life of ^15^O requires a cyclotron in the PET room, which is not available in almost all clinical practices, and PET is too expensive for routine use (Heiss, 2012; Leigh et al., 2018).

MRI-based OEF and CMRO_2_ mapping techniques have recently been developed to evaluate oxygen consumption in tissue using quantitative blood oxygenation level-dependent (BOLD) contrast (He and Yablonskiy, 2007; Yablonskiy et al., 2013a), quantitative imaging of extraction of oxygen and tissue consumption (QUIXOTIC) (Bolar et al., 2011), calibrated BOLD (Davis et al., 1998; Gauthier and Hoge, 2012; Hoge, 2012; Blockley et al., 2013), and quantitative susceptibility mapping (QSM) (Zhang et al., 2015, 2017, 2018; Cho et al., 2018, 2020). In QSM, post-processing of complex 3D multi-echo gradient echo (mGRE) data (de Rochefort et al., 2010), tissue iron (ferritin, diffuse), and blood deoxyheme iron (in venioles, cylinders) can be separated using vascular challenges or prior knowledge (Zhang et al., 2015, 2017, 2018). Recently, OEF mapping can be achieved by combining QSM processing of phase and quantitative blood oxygen level-dependent (qBOLD) modeling of magnitude (Ogawa et al., 1990; Yablonskiy and Haacke, 1994b) of mGRE data without any vascular challenge administration (Cho et al., 2018, 2020), making it ready for routine use in imaging acute ischemic patients. When 3D OEF is multiplied by cerebral blood flow (CBF) from 3D arterial spin labeling (ASL) images, CMRO_2_ can be generated from MRI examinations.

In this work, we report an initial experience using challenge-free OEF mapping from mGRE (Cho et al., 2020) in a cohort of acute and subacute ischemic stroke patients, and compared it with apparent diffusion coefficient (ADC), CBF, and CMRO_2_ mapping.

## Materials and Methods

### Patient Cohort

This was a retrospective study for which written informed consent from patients was waived by the local institutional review board.

A total of 30 consecutive patients with ischemic stroke from January 2014 to January 2015 were recruited according to the following inclusion criteria: (1) the time interval between stroke onset and MRI examination was quantifiable and ranged between 6 h and 15 days; (2) ischemic lesion caused by the stenosis or occlusion of the middle cerebral artery; (3) MRI scan protocol included 3D mGRE and 3D-ASL, in addition to conventional T1 weighted (T1w), T2 weighted (T2w), T2w FLAIR, and DWI sequences; (4) patients did not receive therapy of intravenous thrombolysis or mechanical thrombectomy before the MRI scan; and (5) patients did not show hemorrhage transformation in subsequent MRI scans. A detailed description of the patient characteristics is shown in Table 1.

**TABLE 1:** Patient characteristics and demographics.

	No. of patients	Gender (M/F)	Age (years, mean ± SD)	Time between stroke onset and MRI examination (days, mean ± *SD*)	NIHSS (mean ± *SD*)	Causative subtypes (no. of 1/2/3/4/5*)
Acute	5	4/1	57.6 ± 8.6	0.7 ± 0.4	8.4 ± 7.0	3/0/2/0/0
Early subacute	19	15/4	58.7 ± 11.4	3.7 ± 1.7	7.5 ± 4.0	6/1/4/1/7
Late subacute	6	6/0	52.5 ± 8.9	9.8 ± 2.8	8.2 ± 3.9	3/1/2/0/0

NIHSS, National Institutes of Health Stroke Scale. *Causative subtypes of ischemic stroke (Ay et al., 2005): 1, large artery atherosclerosis; 2, cardioaortic embolism; 3, small-artery occlusion; 4, other causes; 5, undetermined causes.

The included ischemic stroke patients were classified into three groups (Fung et al., 2011) based on the time interval between stroke onset and MRI examination: (1) acute stage (6–24 h, *n* = 5); (2) early subacute stage (1–7 days, *n* = 19); and (3) late subacute stage (7–15 days, *n* = 6) (Table 1).

### MRI Protocol and Image Processing

Brain MRIs were performed on a GE 3.0T scanner (Discovery MR750, GE Healthcare) with a 32-channel head coil. The image protocol consisted of 3D mGRE, 3D-ASL, and conventional sequences (T1w, T2w, T2w FLAIR, and DWI). The acquisition parameters for mGRE were field of view = 24 × 24 cm, TR = 42.8 m, TE1/ΔTE = 4.5/4.9 m, number of TEs = 8, acquisition matrix = 416 × 320, readout bandwidth = 244 Hz/pixel, slice thickness = 2 mm, flip angle = 20°, number of averages = 1, and scan time = 5 min 15 s Parameters for 3D-ASL were field of view = 24 × 24 cm, TR = 4,787 m, TE = 14.6 m, acquisition matrix = 128 × 128, slice thickness = 4 mm, post-labeling delay time = 1,525 m, and number of averages = 3. Parameters for DWI were field of view = 24 × 24 cm, TR = 3,000 m, TE = 71 m, acquisition matrix = 160 × 160, flip angle = 90°, slice thickness = 5 mm, number of averages = 4, *b*-value = 0, 1,000 s/mm^2^. The 3D-ASL and DWI images were sent to a GE workstation (GE Healthcare, AW4.5 workstation) for cerebral blood flow (CBF) and apparent diffusion coefficient (ADC) calculation. QSM was reconstructed from mGRE images using a fully automated zero-referenced morphology enabled dipole inversion (MEDI + 0) method with the ventricular cerebrospinal fluid as a zero reference according to the following optimization (Liu et al., 2018):

(1) $$\underset{\chi \,\left(r\right)}{\text{argmin}}{||w\,\left(r\right)\,\left(e^{-i\,S\,M\,V*d*\chi \,\left(r\right)}-e^{-i\,S\,M\,V*b\,\left(r\right)}\right)||}_{2}^{2}\;+\lambda_{1}\,{||M_{G}\,\left(r\right)\,\nabla \chi \,\left(r\right)||}_{1}+\lambda_{2}\,M_{C\,S\,F}\,\left(r\right)\,{\left(\chi \,\left(r\right)-\bar{\chi_{C\,S\,F}}\right)}_{2}^{2}$$

where *SMV* is the spherical mean value operator (radius 5 mm), *w*(*r*) reflects the reliability of the local field *b*(*r*), *M*_*G*_(*r*) is the edge mask, ∇ is the 3D gradient operator, and $\bar{\chi_{C\,S\,F}}$ is the average of χ(*r*) over the mask *M*_*CSF*_. All the images were co-registered to the magnitude image, which is the geometric mean of the magnitude of mGRE along the time direction and has the same space with QSM, using the FMRIB’s Linear Image Registration Tool algorithm (Jenkinson et al., 2002).

To decompose the susceptibility source into cylindrical deoxyheme iron and diffuse susceptibility source, QSM susceptibility sources was modeled as

(2) $$\chi_{Q\,S\,M}\,\left(Y,v,\chi_{n\,b}\right)\;=\left[\frac{\chi_{b\,a}}{\alpha }+\psi_{H\,b}\cdot \Delta \,\chi_{H\,b}\cdot \left(-Y+\frac{1-\left(1-\alpha \right)\cdot Y_{a}}{\alpha }\right)\right]\cdot v\;+\left(1-\frac{v}{\alpha }\right)\cdot \chi_{n\,b}$$

where α is the vein volume fraction assumed to be constant (0.77), ψ_*Hb*_ is the hemoglobin volume fraction (0.0909 for tissue and 0.1197 for vein), and Δχ_*Hb*_ is the susceptibility difference between deoxy- and oxy-hemoglobin (12,522 ppb). The qBOLD model of the magnitude signal |*s*_*j*_| for the jth echo at echo time *j*Δ*TE* in multi-echo magnitude time evolution was (Yablonskiy et al., 2013b)

(3) $$\left|s_{j}\right|=F_{q\,B\,O\,L\,D}\,\left(Y,v,\chi_{n\,b},s^{0},R_{2},j\,\Delta \,T\,E\right)=s^{0}\,e^{-R_{2}\cdot j\,\Delta \,T\,E}\,e^{-v\,f\,\left(\delta \,\omega \,\left(Y,\chi_{n\,b}\right)\cdot j\,\Delta \,T\,E\right)}\,g\,\left(j\,\Delta \,T\,E\right)$$

where f⁢(δ⁢ω⋅T⁢E)=F21⁢([-12];[34,54];-916⁢(δ⁢ω⋅T⁢E)2)-1 with _1_*F*_2_is the generalized hypergeometric function, δω$\left(Y,\chi_{n\,b}\right)=\frac{1}{3}\cdot \gamma \cdot B_{0}\cdot \left[\text{Hct}\cdot \Delta \,\chi_{0}\cdot \left(1-Y\right)+\chi_{b\,a}-\chi_{n\,b}\right]$, and *g* accounts for the macroscopic contributions due to voxel sensitivity function. Here, γ is the gyromagnetic ratio (267.513 MHz/T), *B*_*0*_ is the main magnetic field (3T in our study), Hct is the hematocrit (0.357), Δχ_0_ is the susceptibility difference between fully oxygenated and fully deoxygenated blood (4π× 0.27 ppm) (Yablonskiy and Haacke, 1994a), χ_*ba*_ is the purely oxygenated blood susceptibility (-108.3 ppb), *Y*is the oxygenation, χ_*nb*_ is the non-blood susceptibility, and *v* is the vein blood volume fraction. In determining $O\,E\,F=1-\frac{Y}{Y_{a}}$ with *Y*_*a*_, the arterial oxygen saturation (0.98), the reconstruction problem from the multi-echo data is to combine the QSM value χ of the phase analysis and the qBOLD data fitting of the magnitude analysis using denoising regularization *R*(*Y*,*v*,χ_*nb*_,*s*^0^,*R*_2_) (Cho et al., 2018):

(4) $$\underset{Y,v,\chi_{n\,b},s^{0},R_{2}}{\text{argmin}}\sum_{j}{||\left|s_{j}\right|-F_{q\,B\,O\,L\,D}\,\left(Y,v,\chi_{n\,b},s^{0},R_{2},j\,\Delta \,T\,E\right)||}_{2}^{2}+w\,{||\chi -\chi_{Q\,S\,M}\,\left(Y,v,\chi_{n\,b}\right)||}_{2}^{2}+\lambda \,R\,\left(Y,v,\chi_{n\,b},s^{0},R_{2}\right)$$

The above minimization problem is solved using a strong denoising algorithm called a cluster analysis of time evolution (CAT) where voxels with similar mGRE magnitude time evolutions are assumed to have similar model parameter values, including OEF, and are grouped into a cluster (Cho et al., 2020). For the grouping, X-means, a modified K-means method that provides an optimal number of clusters, was used with the squared Euclidean distance of the magnitude time evolution across echoes as the similarity measurement. CBF maps were used to further generate the CMRO_2_ maps using the equation: CMRO_2_ = CBF × OEF × [H]_*a*_, where [H]_*a*_ = 7.377 μmol/ml is the oxygenated heme molar concentration in the arteriole.

### Image Analysis

The ischemic core region was manually segmented on co-registered DWI and ADC images by an experienced neuroradiologist (7 years of experience), and the contralateral mirror area with a similar size was also drawn. Infarct cores were defined by identifying the hyperintense regions on the DWI maps and confirmed to be hypointense on the corresponding ADC map. The hypoperfused region on CBF was identified by comparison with the contralateral region. A mismatch region between the hypoperfused region on CBF and the ischemic core on DWI/ADC was also segmented when existed. For each patient, OEF, CMRO_2_, CBF, and ADC were measured on a segmented ischemic core region of interest (ROI) as their absolute values. The differences in OEF, CMRO_2_, CBF, and ADC between an ischemic core and its contralateral ROI were also computed using their contrasts or relative values.

### Statistical Analysis

Statistical analyses were performed with SPSS for Windows (version 22.0, Chicago, IL, United States). The differences among the three ischemic stages for absolute OEF, CMRO_2_, CBF, and ADC values and relative OEF, CMRO_2_, CBF, and ADC were performed using one-way analysis of variance (ANOVA), followed by the least significant difference (LSD) multiple comparison *post hoc* tests. A value of *p* < 0.05 was recognized as statistically significant.

## Results

**FIGURE 1:**
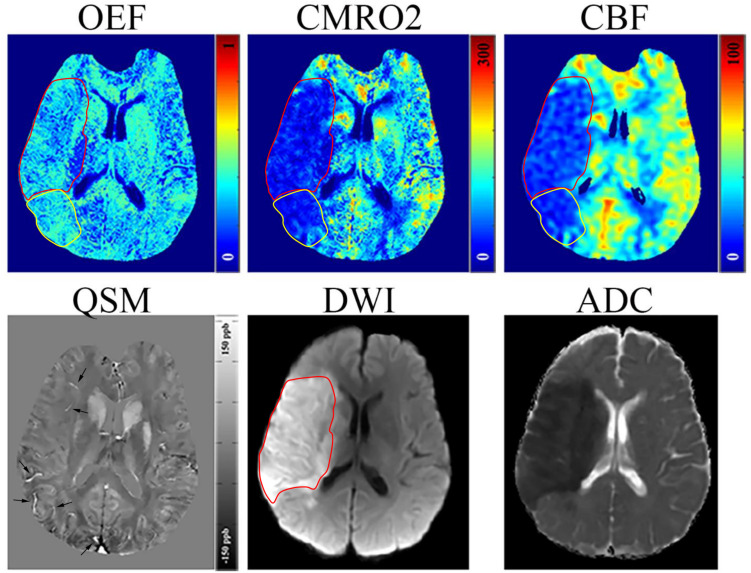
Representative images of an acute ischemic stroke patient with 18 h between MRI examination and stroke symptom onset. The ischemic lesion was caused by the occlusion of the right middle cerebral artery. The ischemic core (red circle) identified on diffusion weighted imaging (DWI) and apparent diffusion coefficient (ADC) maps manifested as a slight decrease (30.38%) in oxygen extraction fraction (OEF) by comparing to the contralateral region (32.12%); some scattered dots within the ischemic core showed a higher OEF. A mismatch (yellow circle) is seen between the cerebral blood flow (CBF) and DWI maps near the ischemic core showing a relatively high OEF (32.15%), slightly higher than the contralateral region, which may represent salvageable ischemic tissue or benign oligemia tissue. Both the ischemic core and mismatch region showed a decrease in cerebral metabolic rate of oxygen (CMRO_2_). On the quantitative susceptibility mapping (QSM) map, some hyperintense veins (arrow) can be seen surrounding the ischemic core.

In three of five acute stroke cases (6, 18, and 24 h from stroke symptom onset), there was a mismatch between the hypoperfused region on CBF and the ischemic core region on DWI/ADC (Figure 1 and Supplementary Figure 1), and the hypoperfused mismatch region had a slightly higher OEF value than the ischemic core region (yellow ROI in Figure 1). The matched region had a similar signal or scattered hypointense signal on OEF when compared to the contralateral region. On CMRO_2_ and CBF maps, the ischemic core region showed a low signal in every stage. There existed no mismatch region in the other two acute stroke cases. The ischemic core region had a decreased ADC for all the five acute cases. The OEF, CMRO_2_, and the relevant CBF, QSM, DWI, and ADC maps of one acute ischemic stroke patient are shown in Figure 1. In early subacute and late subacute stages, the OEF of the ischemic core region manifested as a heterogeneously low signal, compared to the contralateral region.

**FIGURE 2:**
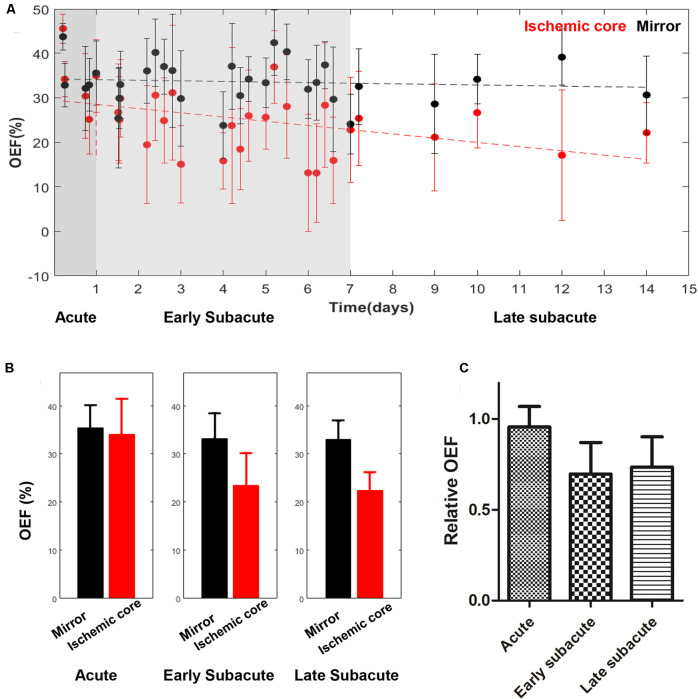
Absolute OEF values in the ischemic core and the contralateral region of the 30 included cases **(A)** and averaged OEF value **(B)**, relative OEF **(C)** in every stage. The absolute OEF value in the ischemic core showed a trend of decrease as the time between symptom onset and MRI examination increased, while OEF values of contralateral regions were relatively stable. Relative OEF also showed a trend of decrease.

**FIGURE 3:**
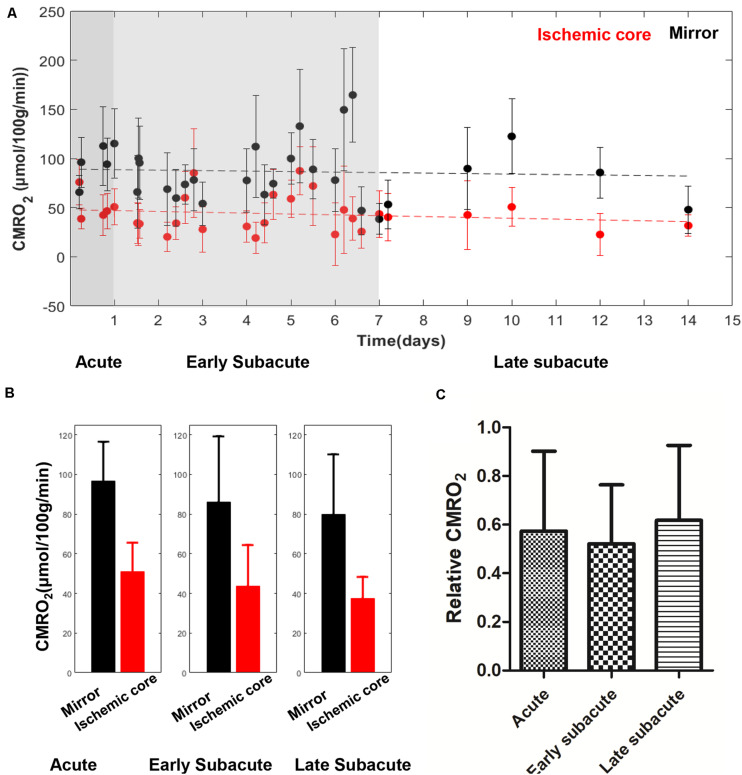
Absolute CMRO_2_ values in the ischemic core and the contralateral region of the 30 included cases **(A)** and averaged CMRO_2_ value **(B)**, relative CMRO_2_ **(C)** in every stage. The absolute CMRO_2_ value in the ischemic core showed a trend of slight decrease with time increase. Relative CMRO_2_ were relatively stable in the three stages.

**FIGURE 4:**
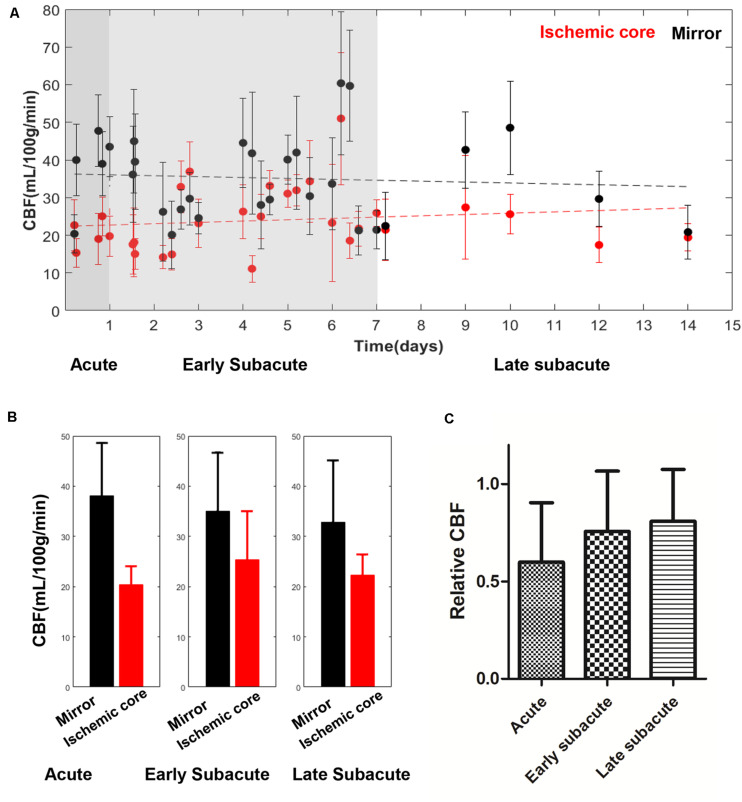
Absolute CBF values in the ischemic core and the contralateral region of the 30 included cases **(A)** and averaged CBF value **(B)**, relative CBF **(C)** in every stage. The absolute CBF value in the ischemic core showed a trend of increase with time increase. Relative CBF also showed a trend of increase.

**FIGURE 5:**
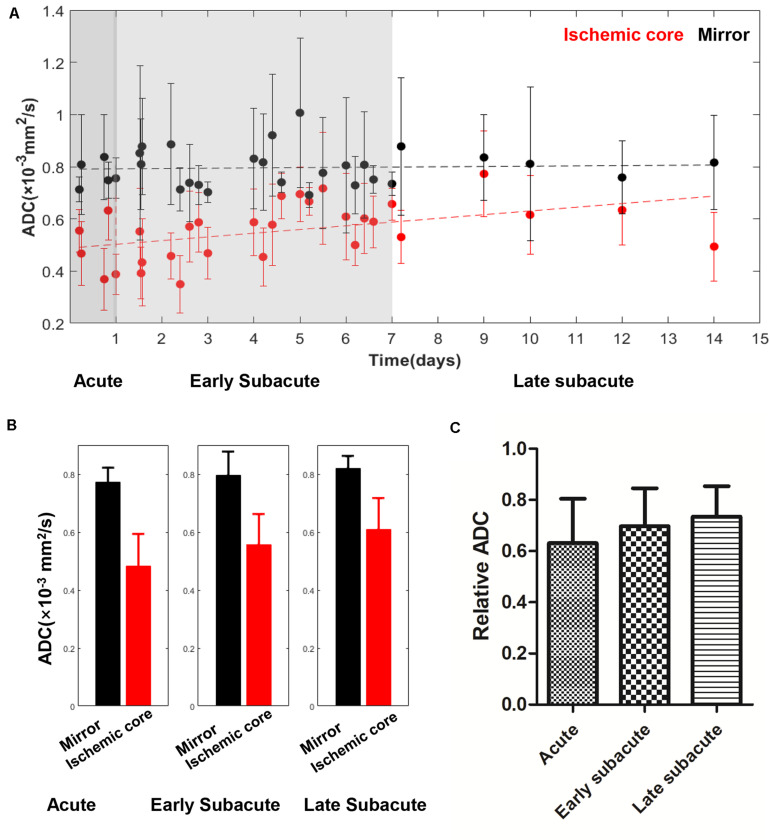
Absolute ADC values in the ischemic core and the contralateral region of the 30 included cases **(A)** and averaged ADC value **(B)**, relative ADC **(C)** in every stage. The absolute ADC value in the ischemic core showed a trend of increase with the time between symptom onset and MRI examination increased, while the ADC value of the contralateral region was relatively stable. Relative ADC also showed a trend of increase.

The absolute OEF, CMRO_2_, CBF, and ADC values of the ischemic core and the contralateral region in each individual case, averaged values, and their differences or relative values in each stage are shown in Figures 2–5. The OEF and CMRO_2_ values in the ischemic core showed a decreasing trend as the time between symptom onset and MRI examination increased (Figures 2, 3, respectively), while the CBF and ADC values showed a slight increasing trend (Figures 4, 5, respectively). There were significant changes among the three stages for the absolute OEF value (*F* = 6.046, *p* = 0.005) in the ischemic core and the relative OEF (*F* = 5.699, *p* = 0.009). *Post hoc* tests showed that the changes in absolute OEF values from acute to early subacute phase (*p* = 0.003), and from acute to late subacute phase (*p* = 0.004) were significant, but not significant from early subacute to late subacute phase (*p* = 0.555); the changes in the relative OEF from acute to early subacute phase (*p* = 0.003) and from acute to late subacute phase (*p* = 0.009) were significant, but not significant from early subacute to late subacute phase (*p* = 0.867). No significant changes among the three stages were found for absolute and relative CMRO_2_, CBF, and ADC values (all values of *p* > 0.05) (Table 2).

**TABLE 2:** The absolute OEF, oxygen extraction fraction; CMRO_2_, cerebral metabolic rate of oxygen; CBF, cerebral blood flow and ADC, apparent diffusion coefficient values of ischemic core, and the relative OEF, CMRO_2_, CBF, and ADC in different stages.

	Absolute OEF (%)	Relative OEF	Absolute CMRO_2_ (μmol/100 g/min)	Relative CMRO_2_	Absolute CBF (ml/100 g/min)	Relative CBF	Absolute ADC (× 10^–3^mm^2^/s)	RelativeADC
Acute	34.03 ± 7.51	0.96 ± 0.11	50.82 ± 14.74	0.57 ± 0.33	20.36 ± 3.70	0.60 ± 0.31	0.48 ± 0.11	0.63 ± 0.17
Early subacute	23.56 ± 6.84	0.70 ± 0.17	43.63 ± 21.32	0.52 ± 0.24	25.27 ± 9.98	0.76 ± 0.31	0.55 ± 0.11	0.70 ± 0.15
Late subacute	21.73 ± 3.82	0.68 ± 0.13	38.54 ± 9.91	0.62 ± 0.31	22.86 ± 4.03	0.81 ± 0.27	0.59 ± 0.11	0.74 ± 0.13
*F, P* value	6.046, 0.005*	5.699, 0.009*	0.585, 0.564	0.316, 0.732	0.733, 0.490	0.738, 0.488	1.508, 0.239	0.734, 0.489

*Significant difference among three different stages using the one-way analysis of variance (ANOVA).

## Discussion

Our preliminary results demonstrate that challenge-free oxygen extraction fraction (OEF) mapping can be performed in a clinical setting. OEF is the most sensitive imaging for measuring tissue functional changes in stroke stages compared to cerebral blood flow (CBF), apparent diffusion coefficient (ADC), and cerebral metabolic rate of oxygen consumption (CMRO_2_). The challenge-free OEF mapping is achieved from quantitative susceptibility mapping (QSM) and quantitative blood oxygen level-dependent (qBOLD) modeling of 3D multi-echo gradient echo data, using cluster analysis of time evolution (CAT) to denoise QSM + qBOLD estimation of OEF. Challenge-free OEF mapping can be used to assess vital oxygen metabolism information of the ischemic tissue for comprehensive stratification of stroke therapy.

The current guidelines (Powers et al., 2018) in selecting eligible acute ischemic stroke patients to receive intravenous thrombolysis therapy are time dependent: the patient should be treated within 3 and 4.5 h of ischemic stroke symptom onset. Now, several studies (Sobesky et al., 2005; Heidenreich et al., 2008; Heiss and Zaro Weber, 2017; Zaro-Weber et al., 2017; Leslie-Mazwi et al., 2018) have reported that identifying the existence of ischemic penumbra using MRI techniques, such as PWI/DWI mismatch approach, can prolong the time window to 24 h, or help change the treatment plan for intra-arterial thrombectomy (Albers et al., 2018). The ischemic penumbra region (Wu et al., 2018), which has preserved neuronal integrity, but impaired function due to hypoperfusion, can recover completely if it receives blood supply in a timely manner but will die otherwise. In the small patient cohort of this study, some of the acute ischemic stroke patients were found to have a slightly elevated OEF in some scattered areas of the ischemic core compared to the contralateral region. This implies that the ischemic core may contain some penumbral tissues even at 4.5 h after the stroke onset.

The CBF/DWI mismatch region in three acute cases was found to have a higher OEF than the ischemic core. The elevated OEF indicates that the ischemic tissue is probably struggling to survive by maintaining energy consumption under reduced blood flow. This information is useful in addressing the ASL-CBF underestimation of the hypoperfused ischemic tissue, due to the presence of a prolonged transit delay resulting from arterial occlusion (Wolf and Detre, 2007; Wang et al., 2012; Bivard et al., 2014). The CBF/DWI mismatch region is difficult to interpret, as it may correspond to ischemic penumbra that benefits from timely revascularization or benign oligemia that does not progress to infarction and does not benefit from thrombotic therapy (Leigh et al., 2018; Wu et al., 2018). The risks of thrombolysis or intra-arterial thrombectomy may outweigh the benefits, if the ischemic core has a substantially decreased OEF, such as in case 5 in Supplementary Figure 1. Therefore, the challenge-free QSM + qBOLD-based OEF mapping that can be performed in clinical settings to assess tissue viability may be included in the routine MRI of ischemic stroke patients for comprehensive evaluation in therapy decision making.

OEF mapping captured the metabolic changes in different ischemic stages that are difficult to detect in CBF and ADC mapping. As the time between stroke symptom onset and MRI examination prolongs, OEF decreases, indicating that the ischemic tissue becomes functionally impaired without timely reperfusion, ending with cell death, consistent with the dynamic process of the pathophysiological change (Dirnagl et al., 1999; Tian et al., 2016). Our observed data on the trend of CBF increase with stroke onset time is consistent with the cerebral perfusion temporal changes measured on CT (Yang et al., 2015), usually as a result of the formation of collateral flow surrounding the ischemic core; a few acute/early subacute cases of CBF at ischemic cores larger than contralateral regions (Figure 4) may reflect the extremes of collateral vascular remodeling or measurement errors. The available CBF map multiplied by the OEF map generates the CMRO_2_ map, which in our results showed a relatively homogenous low signal. CMRO_2_ errors might be mainly driven by the underestimation of ASL–CBF, which was, in part, caused by the short postlabel time (1,525 min) used in our study (Zaharchuk, 2011; Wang et al., 2012; Bivard et al., 2014) and, consequently, were not as sensitive as OEF in detecting metabolic changes in ischemic stroke stages. Accurate computation of CMRO_2_ requires improvements in the accuracy of CBF mapping.

The CAT algorithm based on X-means machine learning has made QSM + qBOLD post-processing of the available multi-echo gradient echo MRI (mGRE) data for OEF mapping robust against noise. This CAT QSM + qBOLD OEF mapping eliminates assumptions in and, therefore, is theoretically more accurate than other OEF mapping methods (Kudo et al., 2016; Uwano et al., 2017; Zhang et al., 2017, 2018). QSM (de Rochefort et al., 2010; Wang and Liu, 2015; Liu et al., 2018) has been shown to be highly reproducible (Deh et al., 2015; Spincemaille et al., 2019), and recent technical developments in preconditioning for optimization execution (Liu et al., 2016) and in phase unwrapping (Dong et al., 2015) promise to make QSM robust even in brain regions near the air–tissue interface. The challenge of qBOLD prone to noise is effectively addressed by the CAT algorithm. The OEF map is generated from mGRE within 25 min on a standard desktop computer, which can be further shortened to a few minutes by optimizing the processing codes and using more powerful computers. Therefore, rapid OEF mapping can be included in routine clinical MRI protocols to accurately evaluate tissue viability in acute ischemic stroke patients, and upon further validation, would help patient management stratification for a timely beneficial therapy.

There are some limitations in this study. The small number of patients, particularly in the acute phase, only allowed a limited demonstration of the broad possible manifestations of the metabolic changes of stroke. The results here may not be generalized without further studies in larger cohorts of stroke patients. The delineation of penumbra tissue at risk, which is of great importance in guiding therapy stratification, could only be shown in three patients. The patients in this cross-section study only had one MRI, and there was an imbalanced gender distribution. Therefore, the benefits of the presented OEF mapping method illustrated here should be understood with caution. Future studies including more acute cases and longitudinal MRI scans are needed to validate our preliminary results. Finally, ischemic stroke patients with hemorrhagic transformation were not included. As further improvements are being developed to account for the strong susceptibility sources of hemorrhages for accurate OEF estimation, OEF mapping using the method described here should become feasible in these patients.

In conclusion, the challenge-free OEF mapping using CAT QSM + qBOLD modeling of mGRE data can be performed in clinical practice to assess oxygen metabolic information, which is helpful in evaluating tissue viability in acute ischemic stroke patients.

## Data Availability Statement

The datasets generated for this study are available on request to the corresponding author.

## Ethics Statement

The studies involving human participants were reviewed and approved by the Institutional Review Board of Tongji Hospital, Tongji Medical College, Huazhong University of Science and Technology, Wuhan, China. Written informed consent for participation was not required for this study in accordance with the national legislation and the institutional requirements.

## Author Contributions

SZ, WZ, and YW were responsible for the study concepts and design. SZ, AG, WZ, and YW were responsible for literature research. SZ, AG, and WZ were responsible for the clinical studies. SZ, JC, TN, and PS were responsible for the statistical analysis. All authors were guarantors of integrity of the entire study and responsible for the experimental studies and data analysis, manuscript preparation and editing, and final approval.

## Conflict of Interest

QSM post-processing software has been developed by Medimagemetric LLC, a Cornell spinoff company that YW owns shares. AG reports non-financial support from GE Healthcare and non-financial support from Siemens Medical Solutions USA, Inc., outside the submitted work. The remaining authors declare that the research was conducted in the absence of any commercial or financial relationships that could be construed as a potential conflict of interest.
