# Supplementary material for: Initial Experience of Challenge-Free MRI-Based Oxygen Extraction Fraction Mapping of Ischemic Stroke at Various Stages: Comparison With Perfusion and Diffusion Mapping
Source: Front Neurosci. 2020 Sep 16;14:535441. doi: 10.3389/fnins.2020.535441 (PMC7525031; doi:10.3389/fnins.2020.535441)
Supplement: Supplementary file 1 [file Data_Sheet_1.PDF]

Supplementary Material

Supplementary Figures

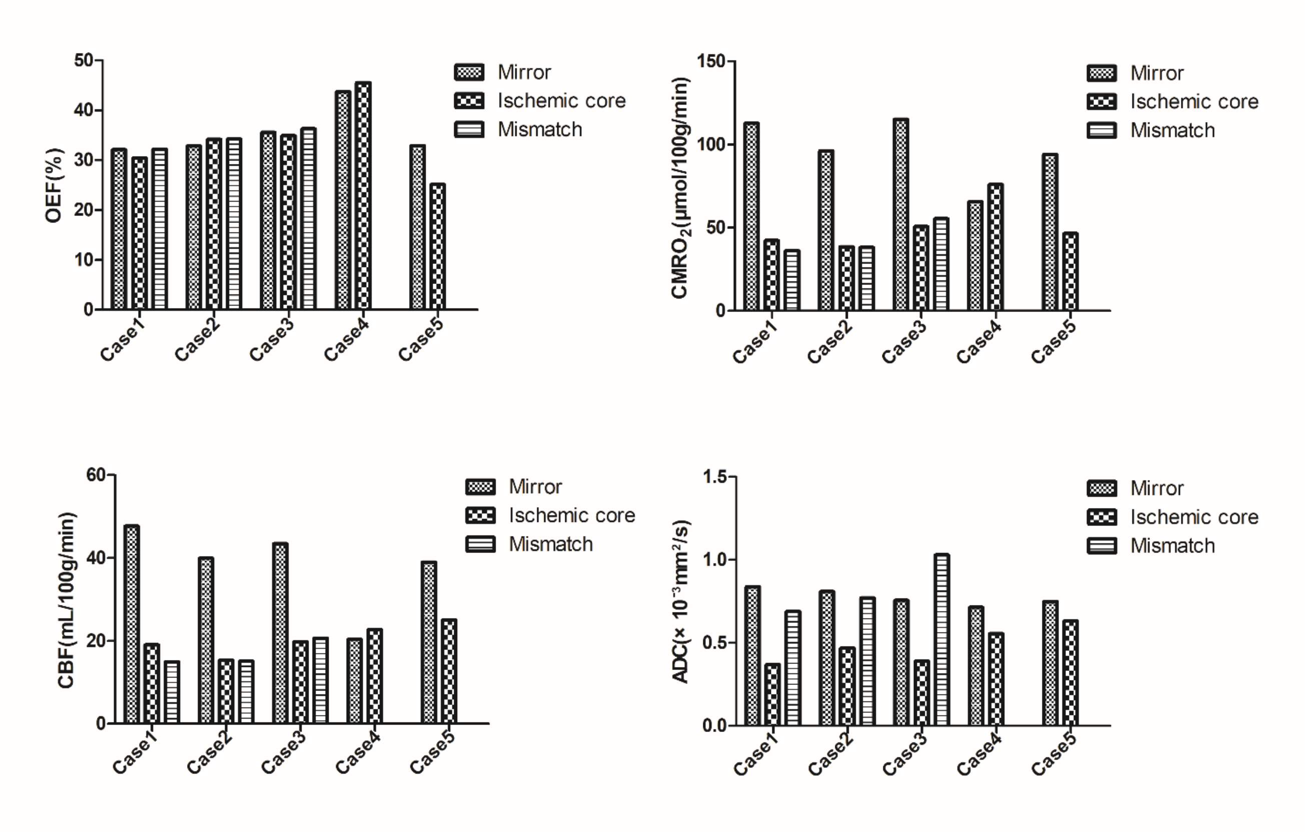

Supplementary Fig 1. The OEF, $CMRO_2$, CBF and ADC values of the five acute ischemic stroke patients (Case 4 and 5 did not have a mismatch region). For case 1-3 (MRI at 18, 6 and 24 hours from stroke symptom onset), the ischemic core had considerable OEF value similar to the contralateral region, and the mismatch region surrounding the ischemic core had a slightly higher OEF value, both the ischemic core and mismatch region had a decreased $CMRO_2$ and CBF. For case 4 (5 hours from stroke symptom onset), the OEF, $CMRO_2$ and CBF in the ischemic core all increased when comparing to the contralateral region, suggesting the ischemic tissue was struggling to survive. For case 5 (20 hours from stroke symptom onset), the OEF value in the ischemic core substantially decreased, suggesting this may not be a candidate benefiting from endovascular thrombectomy.
